# Structures of three polycystic kidney disease-like domains from *Clostridium histolyticum* collagenases ColG and ColH

**DOI:** 10.1107/S1399004714027722

**Published:** 2015-02-26

**Authors:** Ryan Bauer, Katarzyna Janowska, Kelly Taylor, Brad Jordan, Steve Gann, Tomasz Janowski, Ethan C. Latimer, Osamu Matsushita, Joshua Sakon

**Affiliations:** a Department of Chemistry and Biochemistry, University of Arkansas, Fayetteville, AR 72701, USA; b Department of Bacteriology, Okayama University Graduate School of Medicine, Dentistry and Pharmaceutical Sciences, Okayama, Japan

**Keywords:** polycystic kidney disease-like domains, ColG, ColH, *Clostridium histolyticum*

## Abstract

*Clostridium histolyticum* collagenases ColG and ColH are segmental enzymes that are thought to be activated by Ca^2+^-triggered domain reorientation to cause extensive tissue destruction. The collagenases consist of a collagenase module (s1), a variable number of polycystic kidney disease-like (PKD-like) domains (s2a and s2b in ColH and s2 in ColG) and a variable number of collagen-binding domains (s3 in ColH and s3a and s3b in ColG). The X-ray crystal structures of Ca^2+^-bound holo s2b (1.4 Å resolution, *R *= 15.0%, *R*_free_ = 19.1%) and holo s2a (1.9 Å resolution, *R* = 16.3%, *R*_free_ = 20.7%), as well as of Ca^2+^-free apo s2a (1.8 Å resolution, *R* = 20.7%, *R*_free_ = 27.2%) and two new forms of N-terminally truncated apo s2 (1.4 Å resolution, *R* = 16.9%, *R*_free_ = 21.2%; 1.6 Å resolution, *R* = 16.2%, *R*_free_ = 19.2%), are reported. The structurally similar PKD-like domains resemble the V-set Ig fold. In addition to a conserved β-bulge, the PKD-like domains feature a second bulge that also changes the allegiance of the subsequent β-strand. This β-bulge and the genesis of a Ca^2+^ pocket in the archaeal PKD-like domain suggest a close kinship between bacterial and archaeal PKD-like domains. Different surface properties and indications of different dynamics suggest unique roles for the PKD-like domains in ColG and in ColH. Surface aromatic residues found on ColH s2a-s2b, but not on ColG s2, may provide the weak interaction in the biphasic collagen-binding mode previously found in s2b-s3. *B*-factor analyses suggest that in the presence of Ca^2+^ the midsection of s2 becomes more flexible but the midsections of s2a and s2b stay rigid. The different surface properties and dynamics of the domains suggest that the PKD-like domains of M9B bacterial collagenase can be grouped into either a ColG subset or a ColH subset. The conserved properties of PKD-like domains in ColG and in ColH include Ca^2+^ binding. Conserved residues not only interact with Ca^2+^, but also position the Ca^2+^-interacting water molecule. Ca^2+^ aligns the N-terminal linker approximately parallel to the major axis of the domain. Ca^2+^ binding also increases stability against heat and guanidine hydrochloride, and may improve the longevity in the extracellular matrix. The results of this study will further assist in developing collagen-targeting vehicles for various signal molecules.

## Abstract 2

The surface properties and dynamics of PKD-like domains from ColG and ColH differ.

## 1. Introduction   

**Figure 1:**
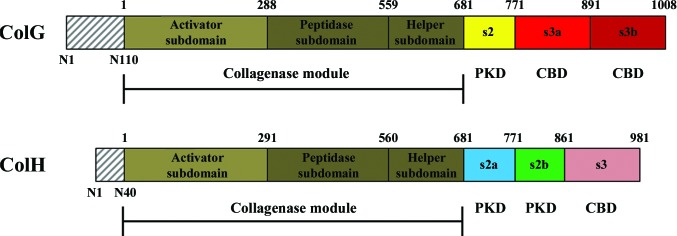
Domain map of collagenases ColG and ColH from *C. histolyticum*. The pre-pro-peptide (grey hatching) is cleaved from the mature enzyme and indicated by sequence numbering N1–N110 (ColG) and N1–N40 (ColH). The collagenase module is composed of an activator subdomain (olive) and peptidase subdomain (dark olive) that is accompanied by a helper subdomain. The PKD-like domain(s) (yellow for ColG; cyan and green for ColH) connect the collagenase module to the C-­terminal CBD(s) (red for ColG; salmon for ColH) that are responsible for collagen binding.

*Clostridium histolyticum* collagenases are causative agents of gas gangrene. The two classes of collagenase, ColG and ColH, differ in domain structures (s1, s2, s3a and s3b for ColG and s1, s2a, s2b and s3 for ColH; Matsushita *et al.*, 2001 ▶; Fig. 1 ▶) and work synergistically to degrade collagen fibers (Breite *et al.*, 2011 ▶). The s1 collagenase module belongs to metallopeptidase subfamily M9B. The amino-acid sequences of s2, s2a and s2b resemble the polycystic kidney disease domain (PKD) that was first identified in the polycystic kidney disease protein PKD1 (The International Polycystic Kidney Disease Consortium, 1995 ▶). The C-terminal domains s3a, s3b and s3 are collagen-binding homologues that are a subclass of bacterial pre-peptidase C-terminal domains (PPC superfamily; Wilson *et al.*, 2003 ▶; Philominathan, Koide *et al.*, 2009 ▶; Philominathan *et al.*, 2012 ▶; Bauer *et al.*, 2013 ▶). The collagen-binding segment composed of the PKD-like domain and collagen-binding domain (CBD) is not necessary to degrade gelatin (denatured, non-triple-helical collagen) and acid-solubilized collagen. However, this segment is necessary to degrade insoluble collagen fibers.

Understanding the interaction of Ca^2+^ is significant owing to its role in regulating both stability and enzyme activity in the extracellular matrix (ECM; Philominathan, Matsushita *et al.*, 2009 ▶; Bauer *et al.*, 2013 ▶; Ohbayashi *et al.*, 2013 ▶). Full-length ColH has been shown to undergo Ca^2+^-dependent structural changes as demonstrated using SAXS and limited proteolysis (Ohbayashi *et al.*, 2013 ▶). In ColG, Ca^2+^ triggers the linker between s3a and s3b to undergo secondary-structure transformation from an α-helix to a β-strand to increase collagen affinity (Wilson *et al.*, 2003 ▶; Sides *et al.*, 2012 ▶). Similar to the N-terminal linker structure of s3b (Philominathan, Matsushita *et al.*, 2009 ▶), the N-terminal linker structure of the PKD-like domain is also thought to be Ca^2+^-dependent, and thus high-resolution structures of both apo and holo states of the PKD-like domains are needed in order to elucidate their activation mechanism. Thus far, crystallographic methods have been used to describe apo s1 from ColG (Eckhard *et al.*, 2011 ▶), the holo peptidase subdomains of ColH (Eckhard *et al.*, 2013 ▶), apo and holo s2 (Eckhard *et al.*, 2011 ▶, 2013 ▶), apo and holo s3b of ColG (Wilson *et al.*, 2003 ▶) and holo s3 of ColH (Bauer *et al.*, 2013 ▶). In the apo s2 structure, however, the conserved Pro688 near the Ca^2+^-binding site was mutated to Thr. As a side note, we use the amino-acid sequence numbering for the mature enzymes. The numbering for s2b and s2a accounts for the cleavage of a 40-amino-acid pre-pro-peptide present in ColH, while the numbering for s2 accounts for the cleavage of a 110-amino-acid pre-pro-peptide present in ColG. In this paper, we describe crystal structures of ColG and, for the first time, ColH PKD-like domains. Thermal and chemical stability differences upon Ca^2+^ binding for the PKD-like domains are also reported.

The collagenolytic mechanism differs between mammalian matrix metalloproteinases (MMPs) and bacterial collagenases (Adhikari *et al.*, 2012 ▶; Duarte *et al.*, 2014 ▶). Unlike bacterial collagenases, MMPs are sequence-specific and are proposed to actively unwind the triple helix (Bertini *et al.*, 2012 ▶; Nagase & Fushimi, 2008 ▶; Welgus *et al.*, 1981*a* ▶,*b* ▶). Meanwhile, each domain in bacterial collagenase is believed to play a unique role in collagenolysis (Fields, 2013 ▶). The C-terminal CBD unidirectionally binds to under-twisted sites in the triple-helical collagenous peptide (Philominathan *et al.*, 2012 ▶; Bauer *et al.*, 2013 ▶). The CBD does not unwind mini-collagen, and hence targeting under-twisted regions of tropocollagen may circumvent the energy barrier required for unwinding the helix. Various roles have been proposed for the PKD-like domains. The PKD-like domain has been shown to swell, but not unwind, collagen fibrils (Wang *et al.*, 2010 ▶). Clostridial PKD-like domains do not bind tightly to collagen fibrils (Matsushita *et al.*, 1998 ▶, 2001 ▶), although s2b has been shown to enhance the ability of s3 to bind to the collagen fiber. S2b-s3 binding is biphasic; the initial low affinity (*K*_d_ = 2.11 × 10^−6^ *M*) leads to higher affinity (*K*_d_ = 3.39 × 10^−7^ *M*) (Matsushita *et al.*, 1998 ▶). The N-terminal collagenase module, s1, has a two-domain architecture that disbands the collagen microfibril into monomeric triple helices and then cleaves the exposed peptide bond preceding the Gly residue (Eckhard *et al.*, 2011 ▶, 2013 ▶). Crystallographic packing analysis of s2 suggested a side-by-side assembly of s1, s2, s3a and s3b that matches the width of the collagen microfibril (Eckhard *et al.*, 2011 ▶; Eckhard & Brandstetter, 2011 ▶). The proposed holo ColG structure is compact; s2 helps to align the active site of s1 with the binding clefts of s3a and s3b. In contrast, the solution envelope of ColH resembles a tadpole (Ohbayashi *et al.*, 2013 ▶), and thus the role of the PKD-like domains in ColH could differ from that in ColG. The work presented here provides a structural framework to better decipher the role of the PKD-like domain.

Despite their detrimental role in bacterial infection, bacterial collagenases and their collagen-binding segments have been investigated for therapeutic applications. A cocktail of *C. histolyticum* ColG and ColH is used in both nonsurgical treatment of Dupuytren’s contracture (Hurst *et al.*, 2009 ▶) and the isolation of pancreatic islets (McCarthy *et al.*, 2011 ▶; Fujio *et al.*, 2013 ▶). Other applications are in preclinical stages (Duarte *et al.*, 2014 ▶). Moreover, fusion proteins consisting of growth factors, cytokines or hormones and the collagen-binding segment s2b-s3 are non-diffusing and long-lasting at wound sites (Nishi *et al.*, 1998 ▶; Akimoto *et al.*, 2013 ▶; Uchida *et al.*, 2013 ▶; Saito *et al.*, 2013 ▶), and hence the binding segments are being developed as drug-delivery vehicles. Therapeutic strategies based on these results are proposed to enhance efficacy by minimizing the quantity of signal molecules necessary and reducing side effects. In contrast, bone distribution of the fusion protein of parathyroid hormone with s3 only (PTH-s3) was efficacious in increasing bone mineral density in osteoporotic models, although fusion proteins of PTH-s2b-s3 demonstrated little efficacy (Ponnapakkam *et al.*, 2012 ▶, 2013 ▶; Ponnapakkam, Katikaneni, Miller *et al.*, 2011 ▶; Ponnapakkam, Katikaneni, Nichols *et al.*, 2011 ▶). When applied to skin, PTH-s3 was efficacious in hair-follicle regeneration in alopecia models (Katikaneni *et al.*, 2012 ▶, 2014 ▶). This study of PKD-like domains is necessary for commercialization and optimization efforts for various drug candidates.

## 2. Methods   

### 2.1. Expression and purification of s2a, s2b and s2   

Expression and purification of each PKD-like domain as a glutathione *S*-transferase (GST)-fusion protein was achieved using previously described methods (Matsushita *et al.*, 2001 ▶).

### 2.2. ^15^N-HSQC NMR characterization of apo s2   

Although stably folded and monodisperse in solution, s2 with its N-terminal linker did not crystallize. ^15^N-enriched apo s2 was produced to measure the dynamics of the protein using NMR. ^15^N-enriched s2 was prepared as described in Philominathan *et al.* (2008 ▶). NMR experiments were performed at 298 ± 0.5 K on a Bruker 700 MHz spectrometer equipped with a cryoprobe. The concentration of the protein used was 0.1 m*M* in 50 m*M* Tris–HCl pH 7.5. In the HSQC spectra, 13 residues could not be identified owing to line broadening (Supplementary Fig. S1). Using the homology-modeled s2 (based on PDB entry 2c4x; Najmudin *et al.*, 2006 ▶), we reasoned that the unobserved HSQC peaks corresponded to a highly dynamic N-terminus that hindered crystallization. Guided by the solution data, 13 residues were truncated from the N-terminus. The truncated s2 crystallized readily.

### 2.3. Crystal structure determination of PKD-like domains   

Initial conditions suitable to grow crystals of apo s2a and holo s2b and two crystal forms of apo s2 were identified by the sitting-drop method using a high-throughput screen (Hampton Research Crystal Screen HT). Subsequent crystallization trials using the initial conditions were carried out using the hanging-drop method. Apo s2a (at a concentration of 30.5 mg ml^−1^) was crystallized from 3 *M* ammonium sulfate, 0.1 *M* MES pH 4.5, 15%(*w*/*v*) PEG 4000 at 290 K, whereas holo s2a (at a concentration of 4.8 mg ml^−1^) was crystallized from 30%(*w*/*v*) PEG 4000, 0.2 *M* MgCl_2_, 0.1 *M* Tris–HCl pH 8.5 at 17°C. These crystals were subsequently soaked in 15%(*w*/*v*) PEG 4000, 0.2 *M* MgCl_2_, 50 m*M* CaCl_2_ , 0.1 *M* Tris–HCl pH 8.5 before initial unit-cell characterization and data collection. Meanwhile, holo s2b (at a concentration of 13.7 mg ml^−1^) was crystallized from 35%(*w*/*v*) PEG 5000, 0.2 *M* ammonium sulfate, 0.1 *M* MES pH 6.5 at 277 K. Both crystal forms of s2 (12.0 mg ml^−1^) were grown from 41–49% 2-methyl-2,4-pentanediol (MPD), 100 m*M* bis-tris pH 5.5, 0.1–0.3 *M* ammonium acetate at 277 K. The in-house X-ray diffraction facility (Rigaku MicroMax-007, Osmic Blue confocal mirrors, Saturn CCD detector) was used for initial characterization of each PKD-like domain crystal, and in the case of the s2a crystals was also used for data collection at 113 K. s2b and s2 crystals were cryocooled and subsequently stored in liquid nitrogen until data collection. Diffraction data were collected at 109 K on the 19-ID beamline of the Advanced Photon Source at Argonne National Laboratory, USA.

Each s2a data set was indexed and scaled using d*TREK (Pflugrath, 1999 ▶), whereas each s2b and s2 data set was indexed and scaled using *HKL*-3000 (Minor *et al.*, 2006 ▶). In each case, a data set truncated to 3 Å resolution was used for molecular replacement using *Phaser* (McCoy *et al.*, 2007 ▶). The PKD-like domain from the carbohydrate-binding module (PDB entry 2c4x) was used as the search model during the structure determination of s2. s2 form I was subsequently used as the search model during structure determination of s2a and s2b. Four molecules were found in the asymmetric unit of the apo s2a crystals, while eight molecules were found in the asymmetric unit of the holo s2a crystals. Meanwhile, two molecules were found in the asymmetric unit of the holo s2b crystals and each form of the apo s2 crystals.

The subsequent structure determination for each model was accomplished using an iterative process of manual adjustments aided by the use of *MIFit* (McRee, 1999 ▶) and refinement using *REFMAC* (Murshudov *et al.*, 2011 ▶). During manual adjustments, *ARP*/*wARP* (Perrakis *et al.*, 1999 ▶) was used to place water molecules. *R*_free_ was lowered for the s2 form I, apo s2a and holo s2a models by utilizing Babinet’s principle for bulk-solvent scaling. In each s2a model, *R*_free_ was also lowered by applying TLS and tight NCS restraints. The s2a models were refined with isotropic *B* factors, whereas the s2 form I and s2b models were refined with anisotropic *B* factors. *PARVATI* (Merritt, 2012 ▶) calculated the anisotropy of s2b and s2 to be 0.5 ± 0.1 and 0.4 ± 0.1, respectively. Isotropic *B* factors would result in an anisotropy of 1.0. Each model exhibited excellent geometry as analyzed by *MolProbity* (Chen *et al.*, 2010 ▶). Full data-collection and refinement statistics are summarized in Table 1 ▶ for s2a, s2b and s2 form I and in Supplementary Table S1 for s2 form II. Alternate conformations are detailed in Supplementary Table S2.

### 2.4. Equilibrium denaturation of PKD-like domains measured using fluorescence spectroscopy   

PKD-like domains share similar topology, and unfolding of each domain was monitored by the change in intrinsic fluorescence of a conserved Trp residue. All experiments were carried out at room temperature using a Hitachi F-2500 fluorimeter with excitation and emission bandwidths at 2.5 and 10 nm, respectively. The excitation wavelength used was 280 nm, and fluorescence emissions were monitored between 300 and 450 nm. For s2b, s2a and s2, λ_max_ for the folded state occurred at 325, 328 and 327 nm, respectively. For each domain, λ_max_ for the denatured state occurred at 350 nm. The ratio of intensity at 350 nm *versus* the intensity at the native-state λ_max_ was used to track the unfolding process. During thermal denaturation trials, the temperature of the protein solution was maintained with a Neslab RTE-110 circulating water bath (Thermo Scientific, Newington, New Hampshire, USA). In the thermal denaturation trials for s2b and s2, the protein concentration was 3 µ*M*. In the chemical denaturation trials, the protein concentration was 1.5 µ*M*. In the case of s2a, the protein concentration for both thermal and chemical denaturation was 5 µ*M*. Each holo PKD-like domain was supplemented with 2 m*M* CaCl_2_, while each apo PKD-like domain was supplemented with 2 m*M* EDTA. In all cases, the protein was diluted in 10 m*M* Tris–HCl pH 7.5, 100 m*M* NaCl. When heat was used as the denaturant, each domain was exposed to temperatures that linearly increased from 280.5 to 363 K in 2.5 K increments. When guanidine hydrochloride (Gu–HCl) was used as the denaturant, each domain was exposed to concentrations of denaturant that increased linearly from 0.0 to 5.8 *M* in 0.2 *M* increments. Δ*G*_HOH_, *C*_M_ and *m* values were calculated as described previously (Philominathan, Matsushita *et al.*, 2009 ▶; Bauer *et al.*, 2013 ▶).

## 3. Results and discussion   

The X-ray crystal structures of Ca^2+^-bound holo s2a (space group *C*2), Ca^2+^-free apo s2a (space group *P*6_1_), Ca^2+^-bound holo s2b (space group *P*2_1_) and two new forms of N-terminally truncated wild-type apo s2 (space groups *P*2_1_ and *P*2_1_2_1_2_1_) are reported for the first time. Of the novel s2a and s2b structures, that of s2b was solved at higher resolution and therefore is described in the most detail. New insights into s2 are subsequently reported.

### 3.1. Overall structure descriptions of apo and holo s2a   

**Figure 2:**
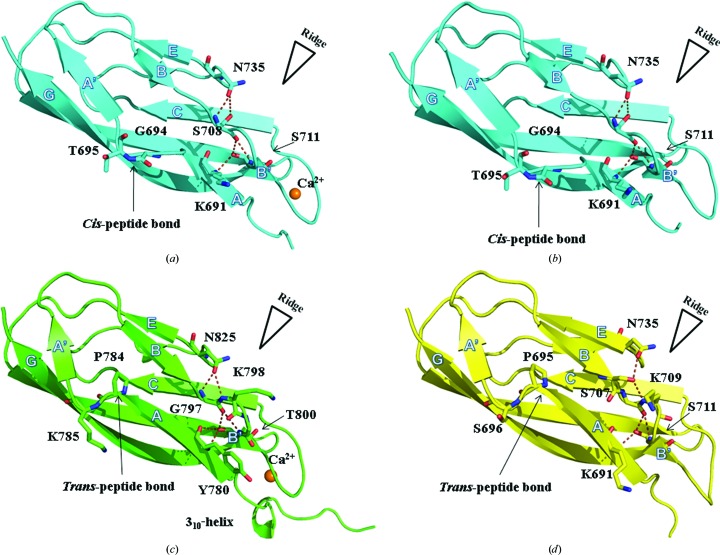
Structural comparison of holo s2a (*a*), apo s2a (*b*), holo s2b (*c*) and apo s2 (*d*). Hydrogen bonds that stabilize β-bulges are highlighted. This figure was prepared using *PyMOL* (Schrödinger).

In the following discussion, holo s2a will be described first (Fig. 2 ▶ *a*). The eight holo s2a molecules are virtually identical (average r.m.s.d. of 0.2 ± 0.1 Å). Here, the molecules spiral along the crystallographic (1, 0, 1) axis. Along this axis, molecule pairs *A* and *G*, *B* and *E*, *C* and *F*, and *D* and *H* are related by NCS translation that results in an off-origin peak that is 63.9% of the origin peak in the Patterson map.

Similar to the molecules in the holo s2a crystal, the four apo s2a molecules are similar (average r.m.s.d. of 0.5 ± 0.2 Å), with molecules *C* and *D* being the most similar (r.m.s.d. = 0.2 Å) and molecules *A* and *D* being the most divergent (r.m.s.d. = 0.8 Å). Molecules *A* and *B*, as well as molecules *C* and *D*, are related by a noncrystallographic twofold. Temperature factors for each structure are relatively high (Table 1 ▶), possibly as a consequence of the high solvent content in the crystal (61.8%).

The holo and apo structures resemble each other (r.m.s.d. = 0.6 ± 0.2 Å). As expected, the most notable difference between the structures occurs near the N-terminus, where Ca^2+^ reorients the interacting residues Asn685 and Ser686. While neither structure could be refined using anisotropic *B* factors, comparison of *B* factors revealed that with the exception of the N-terminal residues, no significant change in *B* factors occurred upon Ca^2+^ binding.

Unlike s2b or s2, s2a truncates β-strand *A* through an approximately 126° rotation of the ψ bond of Ile692. To accommodate the change, Tyr696 packs with Phe706 and is involved in a Δ4 Tyr corner, in which the side-chain hydroxyl group hydrogen-bonds to the backbone four residues prior to it (Hemmingsen *et al.*, 1994 ▶). Interestingly, the Tyr corner also stabilizes the nonprolyl *cis*-peptide bond between Gly694 and Thr695 of s2a that forms the bulge that realigns β-strand *A*′ to interact with β-strand *G* (Fig. 2 ▶ *a* and 2 ▶ *b*). A second bulge between β-strands *B* and *B*′ is stabilized by a hydrogen-bonding network that features a monodentate interaction between Asn735 and Ser708.

### 3.2. Overall structure description of holo s2b   

Similar to holo s2a, the two NCS-related holo s2b structures are virtually identical (r.m.s.d. of 0.4 Å). In the structures, one Ca^2+^ was found near the N-terminal linker (Fig. 2 ▶ *c*). Each holo s2b structure begins at residue 766 and the last residue is 860. The PKD-like domain resembles a V-set Ig fold that lacks two strands (Omit β-strand C′ of the Ig fold corresponds to β-strand D of the PKD-like domain fold). β-strands B, D, and E in the PKD-like domain structures are shorter than the corresponding β-strands of a prototypical V-set Ig fold (PDB entry 1bre; Schormann *et al.*, 1995 ▶), while strands *F* and *G* in the PKD-like domains are longer than the corresponding β-strands in the V-set Ig fold. In the PKD-like domain structures, β-strands A, B, B′ and E form one sheet, while strands A′, C, D, F and G form the opposing sheet. β-strands *A*′ and *G* form a parallel β-sheet, while the remaining strands form antiparallel β-sheets. Meanwhile, β-strand *B* forms a sheet with β-strand *E*, with the exception of Tyr796, which is aligned with β-strand *A*. Given the β-sheet sandwich fold, the PKD-like domain has been predicted to resemble the CBD (Yeats *et al.*, 2003 ▶), although structural alignment of holo s2b with holo s3 (PDB entry 3jqw; Bauer *et al.*, 2013 ▶) suggests little similarity.

Two prominent features are the conserved bulges in the domain that interrupt β-strands *A* and *B* and help to form a ridge along the *ABE* face (Fig. 2 ▶). The first bulge occurs when Pro784 breaks β-strand *A* and pushes the subsequent Lys785 outwards, which in turn leads to an approximately 127° angle between β-strands *A* and *A*′ that also changes the allegiance of β-strand *A*′ to β-strand *G*. The second conserved bulge is introduced by Lys798 and Gly799. This bulge removes the backbone hydrogen-bonding partner of Tyr780 in β-strand *A*. To help stabilize the bulge, the side-chain hydroxyl group of the conserved Thr800 hydrogen-bonds to the backbone amide of Tyr780. To further stabilize the bulge and to position the hydroxyl group of Thr800, the carbonyl O atom of Gly797 hydrogen-bonds to the side-chain hydroxyl group and the main-chain amide of Thr800. The bulge is also stabilized by the conserved Asn825 hydrogen-bonding to the amides of Gly797 and Lys798 (Fig. 2 ▶ *c*). The second bulge helps to form a prominent ridge. Surface-exposed aromatic residues are found on both sides of the ridge and are discussed later.

Temperature factors for both NCS-related structures are low (the average *B* factor for molecule *A* is 11.7 Å^2^ and that for molecule *B* is 9.9 Å^2^). Anisotropic *B*-factor analyses using the *Anisotropic Network Model* (*ANM*) web server (Eyal *et al.*, 2006 ▶) showed that the main chain is mostly isotropic and that potential correlated movement occurs exclusively at the N-terminal linker. The calcium coordination for both holo s2b and holo s2a is described in detail in a later section.

### 3.3. Structure descriptions of apo s2   

Despite being solved in two crystal forms, the crystal structures of forms I and II of apo s2 from ColG are similar. Each asymmetric unit contains two noncrystallographic twofold symmetry-related molecules. All four molecules are virtually identical to each other (r.m.s.d. of <0.5 Å). Each structure begins at residue 685, although the first two residues (Gly-Ser) are remnants from GST-tag cleavage. The last residue is either 770 or 771. The NCS-related molecules are stabilized by antiparallel-type intermolecular interactions between β-strands *A*. Comparison of our apo s2 structures with the previously solved apo s2 structure (PDB entry 2y72; Eckhard *et al.*, 2011 ▶), in which the conserved Pro688 is mutated, showed that the N-terminal mutation pushes the N-terminus out by 3 Å at the C^α^ atom of Ala687. Furthermore, while the residues that make up the previously described bulges are conserved, the interaction pattern differs slightly from the pattern found in s2a and s2b. Here, the hydroxyl group of Ser707 mediates interaction between the conserved Asn735 and the backbone amides of Gly708 and Lys709 (Fig. 2 ▶ *d*).

### 3.4. Structure-based sequence comparisons of PKD-like domains   

**Figure 3:**
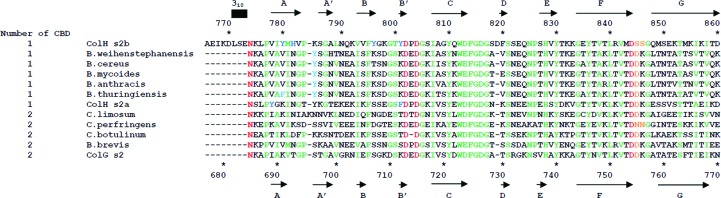
Structure-based sequence alignment of PKD-like domains from M9B. Residues responsible for Ca^2+^ binding and for positioning the Ca^2+^-interacting water, architecturally critical residues and surface aromatic residues are shown in red, orange, green and blue, respectively. Sequence numbering and secondary-structure positions for s2b are shown at the top of the figure. Secondary-structure positions for the s2 structure are similar, although the 3_10_-­helix is absent. Sequence numbering for s2a and s2, as well as secondary-structure positions for s2a, are shown at the bottom of the figure. Sequence alignment was aided by the use of *ClustalW*2 (Thompson *et al.*, 1994 ▶).

Sequence comparison of divergent PKD-like domains revealed conserved residues that are essential for the overall fold and for Ca^2+^ chelation. The residues involved in Ca^2+^ chelation will be discussed in the next section. Peptidase M9 family members are all thought to be collagenases and consist of subfamilies A (*Vibrio*) and B (*Bacillus* and *Clostridium*). M9A enzymes lack CBDs, and consequently may utilize different approaches to collagenolysis. Therefore, this section will discuss M9B-derived PKD-like domains exclusively, and will utilize s2b numbering. The PKD-like domains found in M9B enzymes share two conserved clusters of residues (Fig. 3 ▶). The first conserved stretch, ^802^D*x*DG*x* I*xx*Y*x*WDFGDG^817^, contains β-strand *C* (the underlined residues form the β-strand). This stretch is conserved for its Ca^2+^-binding and architectural importance. The first two Asp residues chelate Ca^2+^. The invariant Tyr810 is accommodated by the second β-bulge. Asp813 is sometimes replaced by an acidic Glu residue. The side chain of Glu may easily fulfill the role of the side chain of Asp813, which terminates β-strand *C* and stabilizes the subsequent sharp turn by hydrogen-bonding to the amide of Gly815. Phe814 stacks against the conserved His828 so that the imidazole ring can also form a salt bridge with the conserved Asp816. Gly817 allows Asp816 to also stabilize the turn by hydrogen-bonding to the amide of Ser818. Although a sharp turn follows β-strand *C* in all PKD-like domains, the type of turn is different. In s2b, the insertion of Asp819 results in the region adopting an ω-loop (*i*→*i* + 10). Both s2a and s2 lack Asp819 in this stretch, and subsequently each is involved in an α-turn (*i*→*i* + 4) that forms a β-hairpin. The second conserved stretch, ^825^NP*x* H*x*Y *xxx* G*x*Y*x*V*x*L*x*V*x*D *xx*G^847^, forms β-strands *E* and *F*. Tyr830 and Tyr836 stack against each other to stabilize the interactions between the sheets. Tyr836 further stabilizes the sheets by forming a Δ4 Tyr corner. Asp844 is responsible for one of the axial interactions with Ca^2+^. The β-strands also wrap around the conserved turn and β-strand *C* to form the most unique feature of the PKD-like domain. Gly847 allows β-strands *F* and *G* to be separated by a β-turn (*i*→*i* + 3).

### 3.5. Ca^2+^ chelation in s2a and s2b   

**Figure 4:**
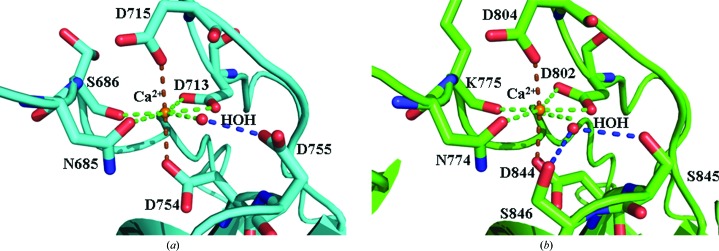
Ca^2+^ coordination in s2a (*a*) and s2b (*b*). Seven O atoms from five residues and one water molecule coordinate to Ca^2+^ in a pentagonal bipyramidal geometry. Pentagonal base interactions are indicated using brown dashes, while axial interactions are indicated using yellow dashes. Residue-to water interactions are indicated with blue dashes. Either one aspartate (s2a) or adjacent serines (s2b) are responsible for positioning the water molecule along the pentagonal base. This figure was prepared using *PyMOL* (Schrödinger).

The Ca^2+^ coordinations in s2a, s2b and s2 are virtually identical to each other. Since holo s2 has been described, this section describes the binding sites in s2a and s2b in detail. One calcium-binding site with a pentagonal bipyramidal geometry was identified near the N-terminus in each domain (Fig. 4 ▶). The pentagonal base is composed of OD1 of Asn685, the main-chain carbonyl of Ser686, OD1 and OD2 of Asp713 and a water molecule, while the axial positions are filled by OD1 of Asp715 and OD2 of Asp754. The Ca—O bond distances and planar deviations amongst the O atoms responsible for the pentagonal base (Table 2 ▶) are similar to the values found in clostridial CBDs (Bauer *et al.*, 2013 ▶). The Ca^2+^ coordination geometry in PKD-like domains has been described as octahedral (Najmudin *et al.*, 2006 ▶; Eckhard & Brandstetter, 2011 ▶), although our results demonstrate that a water is involved in forming a pentagonal base. The coordinating water molecule is positioned by OD1 of Asp755 (Fig. 4 ▶ *a*). The calcium coordination in s2 (PDB entry 4aqo; Eckhard *et al.*, 2013 ▶) resembles that in s2a. Both the water and the calcium ion are ordered (*B* factor < 8.1 Å^2^). Based on the sequence alignment (Fig. 3 ▶) of PKD-like domains, residues contributing side-chain inter­actions with calcium are strictly conserved. s2b chelates with a Ca^2+^ ion very similarly, except for the residues that position the water molecule (Fig. 4 ▶ *b*): s2b utilizes OG from both Ser845 and Ser846 in lieu of Asp755 in s2a.

### 3.6. Ca^2+^-induced structural change   

Ca^2+^ chelation appears to align the N-terminal linker approximately parallel to the major axis of the domain (Supplementary Fig. S2). In s2b, Ca^2+^ chelation by Asn774 and Lys775 could stabilize a 3_10_-helix (residues 770–774) that aligns with the cylinder axis. In s2 and s2a, the N-terminal residues are positioned so that the N-terminal linker could also be positioned parallel to the major axis of the domain. The residues prior to Asn685 cannot be observed in the electron density, and consequently the secondary structure of the region remains ambiguous. Structural comparison of the Ca^2+^-binding site between the apo and holo PKD-like domains revealed that Ca^2+^ has a varied influence on the loop between β-strands *B*′ and *C*. The proline positioned between the aspartates equivalent to Asp802 and Asp804 restricts the loop flexibility so that minimal change occurs between the apo and holo states. However, in s2, which does not have a proline positioned between Asp713 and Asp715, the Ca^2+^ interaction moves Asp715 2 Å from the binding site (Supplementary Fig. 2 ▶ *c*).

**Figure 5:**
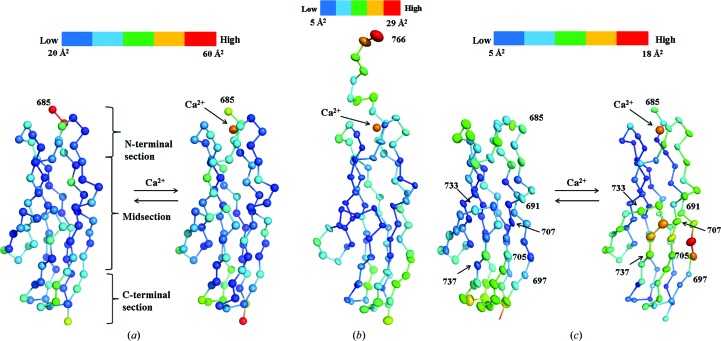
C^α^ *B*-factor changes upon Ca^2+^ binding for s2a (*a*) and s2 (*b*). The C^α^ *B* factors of holo s2b (*c*) are also shown. This figure was prepared using *PyMOL* (Schrödinger).

Overall, the average *B* factors of s2b and s2 are low and the average *B* factors of apo and holo s2a are similar (Table 1 ▶). Despite different symmetry interactions, the *B* factor per-residue trend of 18 PKD-like domains (eight holo s2a molecules, four apo s2a molecules, two holo s2b molecules and four apo s2 molecules) are very similar to each other (Supplementary Figs. S3 ▶ *a*–S3*d*). Holo s2, however, does not follow this trend (Supplementary Fig. S3*e*). Comparison of holo and apo structures revealed a stark contrast between s2a and s2 in the influence of Ca^2+^ (Figs. 5 ▶ *a* and 5 ▶ *c*). Decreases in the C^α^ *B* factor in holo s2 are found in three stretches (Gly698–Ile704, Gly708–Tyr721 and His738–Thr761) that are immediately preceded by stretches in which the *B* factor is higher (Lys691–Thr697, Glu705–Ser707 and Gly733–Val737). In the holo s2 structure the mid-section became more flexible, although both terminal regions became more rigid. Differences in crystal packing could account for the reversal in dynamics, although it is possible that the crystal packing is a consequence of dynamic changes. Both termini of holo s2 are pinned down by symmetry-related molecules that could suppress their dynamics, while the mid-section of the barrel lacks the intermolecular interactions observed in the apo state. In apo s2, intermolecular antiparallel β-sheet interactions involving β-strand *A* could suppress dynamics of the region.

In contrast, comparison of the holo s2a and apo s2a structures revealed that Ca^2+^ did not increase the *B* factor of the mid-section (Fig. 5 ▶ *a*). In the s2a structures the termini are the most flexible. As mentioned, the *B*-factor trends in all holo and apo s2a structures are similar despite the difference in crystal packing. C^α^ *B* factors for the mid-section of holo s2b are low (Fig. 5 ▶ *b*) and suggest that s2b behaves similarly to s2a. Overall, the starkly contrasting dynamics between ColG-derived and ColH-derived PKD-like domains suggest diverging roles during collagenolysis.

### 3.7. Ca^2+^-induced stability gain of PKD-like domains   

**Figure 6:**
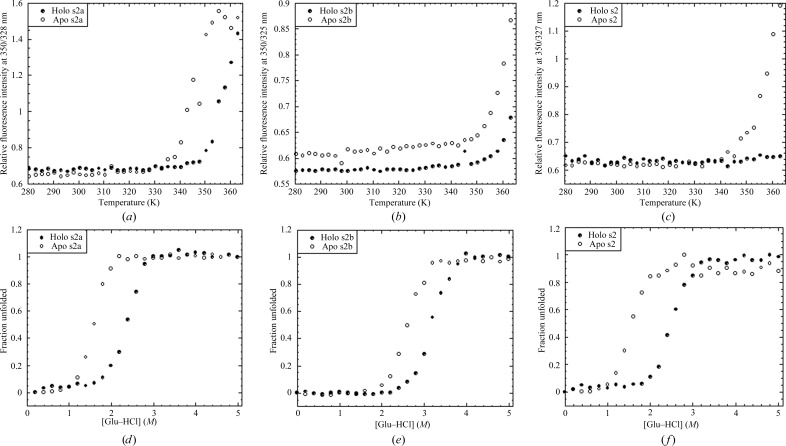
Results of fluorescence-measured equilibrium denaturation of (*a*, *d*) s2a, (*b*, *e*) s2b and (*c*, *f*) s2 in their apo (open circles) and holo (closed circles) forms.

The apo states of s2a, s2b and s2 are thermally stable proteins (*T*_m_ of ∼373 K), but they gain further stability in the presence of Ca^2+^. The fluorescence of Trp812 in s2b was monitored, while in s2a and s2 the fluorescence of Trp723 was monitored. During fluorescence-monitored thermal denaturation, none of the PKD-like domains fully unfolded in the holo state (Figs. 6 ▶ *a*, 6 ▶ *b* and 6 ▶ *c*). Such hyperthermostability has also been observed for the holo states of s3 (Bauer *et al.*, 2013 ▶) and s3b (Philominathan, Matsushita *et al.*, 2009 ▶). The stability of both PKD-like domains and CBD may allow prolonged collagenolytic activity in the ECM. Heat is thought to denature proteins by disrupting electrostatic interactions. As such, the conserved hydrogen-bonding network around the bulges may play a strong role in the overall stability of the domains, while the Ca^2+^–O interactions may contribute to increased stability in the holo state.

PKD-like domains consist of a conserved (shown in green in Fig. 3 ▶) well packed core, and are likewise stable against Gu–HCl denaturation. Here, denaturation occurs through a cooperative transition from the folded state to unfolded states (Figs. 6*d*, 6*e* and 6*f*). In contrast to heat, Gu–HCl is thought to denature proteins predominately by disrupting hydrophobic inter­actions (Monera *et al.*, 1994 ▶). Of the three domains, s2b is the most stable (Table 3 ▶). The difference in Δ*G*_H2O_ between the apo and holo states (ΔΔ*G*_H2O_) is approximately the same for all PKD-like domains. In addition to reorienting the N-terminal linker, the proposed Ca^2+^-induced helical base of the N-terminal linker may have a partial capping effect that shields the core against Gu–HCl. It is also well documented that metalloproteins are more stable in the presence of their metal ligand (Kellis *et al.*, 1991 ▶).

In the clostridial collagen-binding domain, Ca^2+^-induced stability could be partially accounted for by a reduction in void volume and an increase in hydrogen bonds (Philominathan, Matsushita *et al.*, 2009 ▶). Analysis of the void volume of PKD-like domains using *CASTp* (Dundas *et al.*, 2006 ▶) revealed that a common cavity located near the N-terminus shrinks. The common cavity located near the C-terminus of the holo and apo pairs of both s2a and s2 curiously remains essentially unchanged upon Ca^2+^ binding. Furthermore, Ca^2+^ binding does not lead to a significant change in hydrogen-bond totals in any of the domains (Supplementary Table S3 ▶). For therapeutic applications, the *in vitro* stability of s2b and s3 may explain the prolonged activity of growth factors and signal molecules when fused to s2b-s3 *in vivo*.

### 3.8. Surface characteristics of ColG and ColH PKD-like domains   

**Figure 7:**
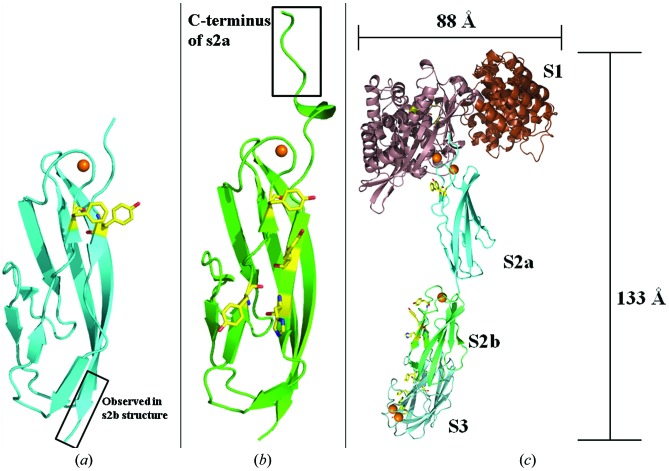
Surface aromatic residues in s2a (*a*) and s2b (*b*). The boxed regions correspond to residues Ala766–Asp770, which are observed in both the s2a and s2b structures and were used to help assemble the full holo ColH structure (*c*). This structure is assembled from the crystal structures of the peptidase domains of s1, s2a, s2b and s3, as well as the homology-modeled activator domain of s1. Homology modeling was accomplished using *SWISS-MODEL* (Biasini *et al.*, 2014 ▶). Surface-exposed aromatic residues of the peptidase domain of s1 and s2a-s2b as well as the conserved collagen-interacting aromatic residues of s3 are shown in yellow. Ca^2+^ is shown as orange spheres. This figure was prepared using *PyMOL* (Schrödinger).

s2a and s2b, unlike s2, contain two and four surface aromatic residues, respectively, that are located on the *ABE* face (Figs. 7 ▶ *a* and 7 ▶ *b*). Interestingly, these residues are also located along the previously mentioned ridge. These residues could be involved in collagen binding given that aromatic residues are found at the hotspot of the collagen-binding pocket in CBD. In s3b, mutations of Tyr970, Ty994 and Tyr996 to Ala greatly reduced binding to collagenous peptide as monitored by surface plasmon resonance (Wilson *et al.*, 2003 ▶). NMR studies also showed that these aromatic residues are involved in collagen binding (Philominathan, Koide *et al.*, 2009 ▶). A structure-based sequence alignment of PKD-like domains (Fig. 3 ▶) suggests that the PKD-like domain of collagenases consisting of only one CBD will likely contain surface aromatic residues. Conversely, the PKD-like domain of collagenases consisting of multiple CBDs, such as s2, appears to have no surface aromatic residues. Collagenases from *B. brevis*, *C. botulinum* and *C perfringens* contain multiple CBDs. Their respective PKD-like domains lack aromatic residues and hence may not directly interact with collagen.

A putative structure of holo ColH can be built from the homology-modeled activator domain of s1 and helical linker (residues 7–301 based on PDB entry 2y50; Eckhard *et al.*, 2011 ▶) and the crystal structures of the peptidase domain (residues 302–681; PDB entry 4ar1; Eckhard *et al.*, 2013 ▶), s2a (residues 685–770), s2b (residues 766–860) and s3 (residues 861–981; PDB entry 3jqw; Bauer *et al.*, 2013 ▶). The overall dimensions (length 133 Å, height 36 Å, width 88 Å) match the tadpole shape observed in the SAXS envelope of holo ColH (Ohbayashi *et al.*, 2013 ▶). In the model, the five-residue overlap between the s2a and s2b structures was superimposed (r.m.s.d. = 1.0 ± 0.1 Å) to assist with formation of the s2a-s2b segment. The aromatic residues mentioned are found on one side of s2a-s2b (Fig. 7 ▶ *c*). In this model, the surface aromatic residues on s2a-s2b may either span across multiple tropocollagen molecules on the surface of the fibril or bind along one tropocollagen molecule when the binding surface of s3 is docked onto the collagen fibril surface. The interactions may serve to prevent the collagen-binding segment from diffusing away after the s3–collagen interaction is transiently broken. Likewise, the domains may provide loose contacts with the collagen fibril that allow the enzyme to scan the fibril surface for optimal regions for tight CBD interaction. In these roles, the PKD-like domain strengthens collagen avidity so that only one CBD is required for collagen binding. The zinc ion involved in activation of a water molecule is approximately 115 Å away from Tyr962 found in the collagen-binding pocket of s3. In this model, the PKD-like domains may also be critical in positioning the catalytic domain with respect to CBD.

### 3.9. Potential role of PKD-like domains in synergistic collagenolysis   

The apparent differences between the ColG-derived PKD-like domain and the ColH-derived PKD-like domains may aid synergistic collagenolysis. The putative holo ColH structure and the structure-based insights into the PKD-like domains allow us to begin to speculate on how ColG and ColH work together to degrade collagen. Currently, it is not known whether any of the clostridial PKD-like domains swell collagen fibers. Both s3 and s3b share a common preference for under-twisted regions of collagen (Bauer *et al.*, 2013 ▶), although ColG and ColH initially cleave different sites in collagen (French *et al.*, 1992 ▶). When digesting the insoluble fiber, ColH is the workhorse (Breite *et al.*, 2011 ▶). The higher collagen affinity observed for s2b-s3 may increase further on the addition of s2a. The increased affinity could hold ColH close to the collagen fibril so that it can slide along the fibril and find vulnerable regions. Meanwhile, holo ColG has been propsed to adopt a compact structure in which the domains of the collagen-bonding segment are aligned by intermolecular β-sheet-type hydrogen-bond interactions (Eckhard *et al.*, 2011 ▶). The tandem CBDs of ColG may allow the enzyme to anchor itself to the most vulnerable region of the fibril. In this context, the spring-like dynamics of s2 may allow it to swell the fibril. The swelled fibril would then expose the interior of the fibril and expose new sites for ColH collagenolysis.

### 3.10. PKD evolution   

Human PKD1 (PDB entry 1b4r; Bycroft *et al.*, 1999 ▶) and PKD-like domains from archaea and bacteria share a high degree of structural similarity that suggests that the fold has laterally transferred across the kingdoms. As expected, s2a, s2b and s2 resemble the *C. thermocellum* endoglucanase PKD-like domain (PDB entry 2c4x; Najmudin *et al.*, 2006 ▶) more closely than either the archaeal surface protein PKD-like domain (PDB entry 1l0q; Jing *et al.*, 2002 ▶) or human PKD1. While the bulge between β-strands *A* and *A*′ appears to be well conserved only in bacterial PKD-like domains, the bulge between β-strands *B* and *B*′ is also conserved in the archaeal PKD-like domain but is not conserved in PKD1. Correspondingly, residues Thr800 and Asn825, which are critical for stabilizing this bulge, are conserved in the archaeal PKD-like domain. Oddly, only Thr800 is conserved in the endoglucanase PKD-like domain. Normally, surface inter­actions are not well conserved, but surprisingly the salt bridge formed between Asp816 and His828 in s2b is found in the archaeal PKD-like domain. Structurally equivalent residues in the endoglucanase PKD-like domain, Asp47 and Tyr60, utilize hydrogen-bonding between OD1 and OD2 of Asp and OH of Tyr in lieu of the salt bridge. In the domains, these interactions serve to stack the β-sheets together and stabilize the region where β-strand *D* of the Ig fold is deleted. The interaction is not found between the equivalent Asp and His residues in the NMR structure of human PKD1, although it should be noted that the NMR structure is derived from main-chain NOE restraints and therefore the side-chain orientations are not experimentally obtained. Thus, these residues may also assist in the interaction of β-strands *C* and *E*. Within the core, Trp812 and Phe795 are conserved throughout the three kingdoms. Our PKD-like domain structures suggest that this Phe residue strengthens the interactions of β-strands *B* and *C* through packing with the strictly conserved Trp and Phe in strand *D*. The residue further supports the barrel architecture through hydrophobic packing with the C-terminal region of the barrel.

**Figure 8:**
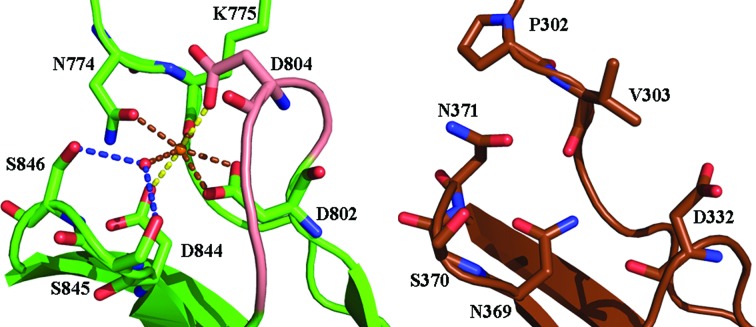
Proposed evolution of the Ca^2+^-binding pocket in bacterial PKD-like domains (holo s2b is shown on the left) from the archaeal PKD-like domain (PDB entry 1loq, shown on the right). This figure was prepared using *PyMOL* (Schrödinger).

Comparison of bacterial holo PKD-like domains with either archaeal or mammalian PKDs suggests that the Ca^2+^-binding site in bacteria evolved from archaea. In addition to the overall structural similarity, five of the seven O atoms that coordinate to Ca^2+^ are present in the archaeal PKD-like domain. The archaeal domain lacks the initial asparagine residue and one of the axial aspartate residues required for Ca^2+^ binding. The Ca^2+^-interacting residues Asn774 and Lys775 of s2b are replaced by Pro302 and Val303 in archaea. In addition to removing an O atom responsible for the pentagonal base, Pro appears to constrain Val303 O to the position occupied by Ca^2+^ (Fig. 8 ▶). The archeal PKD-like domain possesses a bidentate Asp802 equivalent. However, the loop is significantly shortened compared with the bacterial domains, which consequently removes the Asp804 equivalent. It should be noted that the water-positioning residues in s2b appear to be conserved in archaea (Ser845 is conserved, while Ser846 is replaced by asparagine). The mammalian PKD, meanwhile, lacks all of the residues that interact with Ca^2+^.

Comparison of clostridial PKD-like domain structures with V-set kappa light-chain Bence–Jones protein (PDB entry 1bre; Schormann *et al.*, 1995 ▶) as well as with archaeal PKD-like domains and human PKD suggests that the PKD1 domain fold in eukaryotes descended from the simpler Ig fold and may then have spread to archaea. From archaea, the fold spread laterally to bacteria. Characteristics of the V-set Ig fold that are shared with the PKD-like domain fold are the following. (i) The tertiary structure consists of a two-faced β-sheet architecture made up from a well packed hydrophobic core. (ii) β-Strand *A* is broken by a conserved bulge that changes the allegiance of the subsequent β-strand *A*′. (iii) β-Strand *C* contains the conserved tryptophan, and along with β-strand *C*′ (β-strand *D* of the PKD-like domain fold) forms a β-hairpin connected by an approximately *i*→*i* + 8 ω-loop. (iv) The turn leading into β-strand *F* is stabilized by a Δ4 Tyr corner.

## 4. Conclusion   

Comparison of crystal structures of ColG s2 with crystal structures of ColH s2a and s2b suggests that despite common tertiary folds, PKD-like domains can be grouped into two subsets. The subset containing ColH-derived domains exhibits exposed aromatic residues and is found in M9B collagenases with a single CBD. The surface aromatic residues could be involved in secondary interactions that allow weak collagen binding. In contrast, the subset containing s2 is likely to be different; the lack of surface aromatic residues on s2 suggests that the domain is less directly involved in interactions with collagen. Overall, this subset is found in M9B collagenases with multiple CBDs. The unique differences in dynamics and surface characteristics between s2a-s2b and s2 may aid in synergistic collagenolysis.

Meanwhile, the N-terminal linker structure of a PKD-like domain is described for the first time in the holo s2b structure and suggests that Ca^2+^ repositions the linker along the barrel axis. The helical structure of the linker upon Ca^2+^ binding may shorten the distance between s2b and s2a, and may help to account for the previously described proteinase resistance (Ohbayashi *et al.*, 2013 ▶). Lastly, our stability data show that the domains are extremely stable in the presence of physiological Ca^2+^. Structural and stability data are critical for the development of PKD-like domains as part of the site-directed delivery of signal molecules such as growth factors and cytokines.

## Supplementary Material

PDB reference: ColG PKD domain 2, 4jrw

PDB reference: 4tn9

PDB reference: ColH PKD domain 2a, apo, 4u6t

PDB reference: in the presence of calcium, 4u7k

PDB reference: ColH PKD domain 2b, in the presence of calcium, 4jgu

Supporting Information.. DOI: 10.1107/S1399004714027722/mn5081sup1.pdf

## Figures and Tables

**Table 1:** Data-collection and refinement statistics Values in parentheses are for the highest resolution shell.

	Holo s2a	Apo s2a	Holo s2b	Apo s2 form I
Data collection				
X-ray wavelength ()	1.54	1.54	0.97937	0.919
Space group	*C*2	*P*6_1_	*P*2_1_	*P*2_1_
Unit-cell parameters				
*a* ()	102.3	88.3	49.38	25.00
*b* ()	87.6	88.3	38.87	71.76
*c* ()	104.4	123.6	54.66	47.89
()	90.0	90.0	90.0	90.0
()	116.0	90.0	98.43	95.56
()	90.0	120.0	90.00	90.00
Resolution ()	93.81.9 (1.981.91)	76.51.8 (1.811.76)	22.191.42 (1.441.42)	47.671.40 (1.441.40)
No. of reflections	224470	252508	137190	124611
Multiplicity	3.6 (3.1)	4.7 (2.1)	3.5 (2.9)	3.3 (2.4)
Completeness (%)	96.4 (93.8)	99.4 (95.7)	96.7 (85.8)	92.5 (83.7)
*I*/(*I*)	13.8 (3.0)	11.5 (2.3)	33.0 (3.1)	21.3 (2.0)
*R*_meas_ † (%)	5.4 (31.0)	7.5 (37.4)	4.6 (38.5)	8.1 (46.9)
Refinement				
Unique reflections	58720	50932	35993	28786
*R*_cryst_ ‡ (%)	16.3 (25.5)	20.7 (26.8)	15.0 (25.4)	16.9 (28.3)
*R*_free_ § (%)	20.7 (29.7)	27.2 (32.0)	19.1 (33.2)	21.2 (35.6)
Average *B* factor (^2^)				
Chain *A*, main chain	18.9	17.6	9.6	12.5
Chain *A*, side chains	23.3	21.1	13.7	16.5
Chain *B*, main chain	18.9	17.9	8.3	12.5
Chain *B*, side chains	23.5	21.4	12.2	16.8
Chain *C*, main chain	18.9	18.9	N/A	N/A
Chain *C*, side chains	23.6	22.0	N/A	N/A
Chain *D*, main chain	18.7	19.3	N/A	N/A
Chain *D*, side chains	23.1	22.3	N/A	N/A
Chain *E*, main chain	19.0	N/A	N/A	N/A
Chain *E*, side chains	23.5	N/A	N/A	N/A
Chain *F*, main chain	18.7	N/A	N/A	N/A
Chain *F*, side chains	23.0	N/A	N/A	N/A
Chain *G*, main chain	18.8	N/A	N/A	N/A
Chain *G*, side chains	23.1	N/A	N/A	N/A
Chain *H*, main chain	19.0	N/A	N/A	N/A
Chain *H*, side chains	23.5	N/A	N/A	N/A
Solvent	46.1	42.2	25.6	31.1
Ramachandran statistics				
Favored (%)	98.5	99.4	98.9	99.4
Additionally allowed (%)	1.5	0.6	1.1	0.6
Outliers (%)	0	0	0	0

† *R*_meas_ = 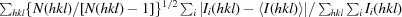.

‡ *R*_cryst_ = 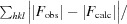 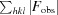 for the 95% of reflection data used for refinement.

§ *R*_free_ = 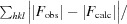 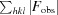 for the 5% of reflection data excluded from refinement.

**Table 2:** CaO bond distances and planar deviations for holo s2b and holo s2a

	Residue	Atom	Distance ()	Planar deviation ()
s2b				
Molecule *A*	HOH	O	2.49	0.09
	Asn774	OD1	2.39	0.20
	Lys775	O	2.41	0.27
	Asp802	OD1	2.42	0.38
	Asp802	OD2	2.44	0.22
	Asp804: axial	OD1	2.34	N/A
	Asp844: axial	OD2	2.42	N/A
Molecule *B*	HOH	O	2.40	0.14
	Asn774	OD1	2.37	0.24
	Lys775	O	2.38	0.28
	Asp802	OD1	2.46	0.38
	Asp802	OD2	2.49	0.19
	Asp804: axial	OD1	2.34	N/A
	Asp844: axial	OD2	2.39	N/A
s2a				
Average of molecules *A* *H*	HOH	O	2.53 (5)	0.03 (2)
	Asn685	OD1	2.52 (6)	0.10 (3)
	Ser686	O	2.43 (6)	0.14 (3)
	Asp713	OD1	2.51 (3)	0.15 (3)
	Asp713	OD2	2.50 (6)	0.09 (4)
	Asp715: axial	OD1	2.42 (3)	NA
	Asp754: axial	OD2	2.40 (5)	NA

**Table 3:** Stability parameters for GuHCl denaturation of PKD-like domains

	Holo s2b	Apo s2b	Holo s2a	Apo s2a	Holo s2	Apo s2
*G* (kcalmol^1^)	9.8	6.9	7.8	4.9	6.0	4.3
*m* (kcalmol^1^ *M* ^1^)	3.1	2.6	3.6	3.1	2.4	2.7
*C*_M_ (*M*)	3.2	2.6	2.2	1.6	2.5	1.6

## References

[bb1] Adhikari, A. S., Glassey, E. & Dunn, A. R. (2012). *J. Am. Chem. Soc.* **134**, 13259–13265. 10.1021/ja212170b PMC4800024 22720833

[bb2] Akimoto, M., Takeda, A., Matsushita, O., Inoue, J., Sakamoto, K., Hattori, M., Kounoike, N. & Uchinuma, E. (2013). *Plast. Reconstr. Surg.* **131**, 717–725. 10.1097/PRS.0b013e3182818b34 23542245

[bb3] Bauer, R., Wilson, J. J., Philominathan, S. T., Davis, D., Matsushita, O. & Sakon, J. (2013). *J. Bacteriol.* **195**, 318–327. 10.1128/JB.00010-12 PMC3553835 23144249

[bb4] Bertini, I., Fragai, M., Luchinat, C., Melikian, M., Toccafondi, M., Lauer, J. L. & Fields, G. B. (2012). *J. Am. Chem. Soc.* **134**, 2100–2110. 10.1021/ja208338j PMC3298817 22239621

[bb5] Biasini, M., Bienert, S., Waterhouse, A., Arnold, K., Studer, G., Schmidt, T., Kiefer, F., Cassarino, T. G., Bertoni, M., Bordoli, L. & Schwede, T. (2014). *Nucleic Acids Res.* **42**, W252–W258. 10.1093/nar/gku340 PMC4086089 24782522

[bb6] Breite, A. G., McCarthy, R. C. & Dwulet, F. E. (2011). *Transplant. Proc.* **43**, 3171–3175. 10.1016/j.transproceed.2011.09.059 22099748

[bb7] Bycroft, M., Bateman, A., Clarke, J., Hamill, S. J., Sandford, R., Thomas, R. L. & Chothia, C. (1999). *EMBO J.* **18**, 297–305. 10.1093/emboj/18.2.297 PMC1171124 9889186

[bb8] Chen, V. B., Arendall, W. B., Headd, J. J., Keedy, D. A., Immormino, R. M., Kapral, G. J., Murray, L. W., Richardson, J. S. & Richardson, D. C. (2010). *Acta Cryst.* D**66**, 12–21. 10.1107/S0907444909042073 PMC2803126 20057044

[bb9] Duarte, A. S., Correia, A. & Esteves, A. C. (2014). *Crit. Rev. Microbiol.*, 10.3109/1040841X.2014.904270. 10.3109/1040841X.2014.904270 24754251

[bb10] Dundas, J., Ouyang, Z., Tseng, J., Binkowski, A., Turpaz, Y. & Liang, J. (2006). *Nucleic Acids Res.* **34**, W116–W118. 10.1093/nar/gkl282 PMC1538779 16844972

[bb11] Eckhard, U. & Brandstetter, H. (2011). *Biol. Chem.* **392**, 1039–1045. 10.1515/BC.2011.099 21871007

[bb12] Eckhard, U., Schönauer, E. & Brandstetter, H. (2013). *J. Biol. Chem.* **288**, 20184–20194. 10.1074/jbc.M112.448548 PMC3711286 23703618

[bb13] Eckhard, U., Schönauer, E., Nüss, D. & Brandstetter, H. (2011). *Nature Struct. Mol. Biol.* **18**, 1109–1114. 10.1038/nsmb.2127 PMC3191118 21947205

[bb14] Eyal, E., Yang, L.-W. & Bahar, I. (2006). *Bioinformatics*, **22**, 2619–2627. 10.1093/bioinformatics/btl448 16928735

[bb15] Fields, G. B. (2013). *J. Biol. Chem.* **288**, 8785–8793. 10.1074/jbc.R113.451211 PMC3610953 23430258

[bb16] French, M. F., Bhown, A. & Van Wart, H. E. (1992). *J. Protein Chem.* **11**, 83–97. 10.1007/BF01025095 1325154

[bb17] Fujio, A., Murayama, K., Yamagata, Y., Watanabe, K., Imura, T., Inagaki, A., Ohbayashi, N., Shima, H., Sekiguchi, S., Fujimori, K., Igarashi, K., Ohuchi, N., Satomi, S. & Goto, M. (2013). *Transplantation*, **96**, S141–S142.

[bb18] Hemmingsen, J. M., Gernert, K. M., Richardson, J. S. & Richardson, D. C. (1994). *Protein Sci.* **3**, 1927–1937. 10.1002/pro.5560031104 PMC2142652 7703839

[bb19] Hurst, L. C., Badalamente, M. A., Hentz, V. R., Hotchkiss, R. N., Kaplan, F. T., Meals, R. A., Smith, T. M. & Rodzvilla, J. (2009). *N. Engl. J. Med.* **361**, 968–979. 10.1056/NEJMoa0810866 19726771

[bb20] Jing, H., Takagi, J., Liu, J.-H., Lindgren, S., Zhang, R.-G., Joachimiak, A., Wang, J.-H. & Springer, T. A. (2002). *Structure*, **10**, 1453–1464. 10.1016/s0969-2126(02)00840-7 12377130

[bb21] Katikaneni, R., Ponnapakkam, T., Matsushita, O., Sakon, J. & Gensure, R. (2014). *Anticancer Drugs*, **25**, 30–38. 10.1097/CAD.0b013e3283650bff PMC4005392 24025564

[bb22] Katikaneni, R., Ponnapakkam, T., Suda, H., Miyata, S., Sakon, J., Matsushita, O. & Gensure, R. C. (2012). *Int. J. Cancer*, **131**, E813–E821. 10.1002/ijc.27379 PMC3693573 22130912

[bb23] Kellis, J. T. Jr, Todd, R. J. & Arnold, F. H. (1991). *Nature Biotechnol.* **9**, 994–995. 10.1038/nbt1091-994 1369354

[bb24] Matsushita, O., Jung, C.-M., Minami, J., Katayama, S., Nishi, N. & Okabe, A. (1998). *J. Biol. Chem.* **273**, 3643–3648. 10.1074/jbc.273.6.3643 9452493

[bb25] Matsushita, O., Koide, T., Kobayashi, R., Nagata, K. & Okabe, A. (2001). *J. Biol. Chem.* **276**, 8761–8770. 10.1074/jbc.M003450200 11121400

[bb26] McCarthy, R. C., Breite, A. G., Green, M. L. & Dwulet, F. E. (2011). *Transplantation*, **91**, 137–145. 10.1097/TP.0b013e3181ffff7d PMC3022104 21116222

[bb27] McCoy, A. J., Grosse-Kunstleve, R. W., Adams, P. D., Winn, M. D., Storoni, L. C. & Read, R. J. (2007). *J. Appl. Cryst.* **40**, 658–674. 10.1107/S0021889807021206 PMC2483472 19461840

[bb28] McRee, D. E. (1999). *Practical Protein Crystallography*, 2nd ed. San Diego: Academic Press.

[bb29] Merritt, E. A. (2012). *Acta Cryst.* D**68**, 468–477. 10.1107/S0907444911028320 PMC3322606 22505267

[bb30] Minor, W., Cymborowski, M., Otwinowski, Z. & Chruszcz, M. (2006). *Acta Cryst.* D**62**, 859–866. 10.1107/S0907444906019949 16855301

[bb31] Monera, O. D., Kay, C. M. & Hodges, R. S. (1994). *Protein Sci.* **3**, 1984–1991. 10.1002/pro.5560031110 PMC2142645 7703845

[bb32] Murshudov, G. N., Skubák, P., Lebedev, A. A., Pannu, N. S., Steiner, R. A., Nicholls, R. A., Winn, M. D., Long, F. & Vagin, A. A. (2011). *Acta Cryst.* D**67**, 355–367. 10.1107/S0907444911001314 PMC3069751 21460454

[bb33] Nagase, H. & Fushimi, K. (2008). *Connect. Tissue Res.* **49**, 169–174. 10.1080/03008200802151698 18661336

[bb34] Najmudin, S., Guerreiro, C. I. P. D., Carvalho, A. L., Prates, J. A. M., Correia, M. A. S., Alves, V. D., Ferreira, L. M., Romao, M. J., Gilbert, H. J., Bolam, D. N. & Fontes, C. M. G. A. (2006). *J. Biol. Chem.* **281**, 8815–8828. 10.1074/jbc.M510559200 16314409

[bb35] Nishi, N., Matsushita, O., Yuube, K., Miyanaka, H., Okabe, A. & Wada, F. (1998). *Proc. Natl Acad. Sci. USA*, **95**, 7018–7023. 10.1073/pnas.95.12.7018 PMC22723 9618531

[bb36] Ohbayashi, N., Matsumoto, T., Shima, H., Goto, M., Watanabe, K., Yamano, A., Katoh, Y., Igarashi, K., Yamagata, Y. & Murayama, K. (2013). *Biophys. J.* **104**, 1538–1545. 10.1016/j.bpj.2013.02.022 PMC3617444 23561530

[bb37] Perrakis, A., Morris, R. & Lamzin, V. S. (1999). *Nature Struct. Biol.* **6**, 458–463. 10.1038/8263 10331874

[bb38] Pflugrath, J. W. (1999). *Acta Cryst.* D**55**, 1718–1725. 10.1107/s090744499900935x 10531521

[bb39] Philominathan, S. T., Koide, T., Hamada, K., Yasui, H., Seifert, S., Matsushita, O. & Sakon, J. (2009). *J. Biol. Chem.* **284**, 10868–10876. 10.1074/jbc.M807684200 PMC2667773 19208618

[bb40] Philominathan, S. T., Koide, T., Matsushita, O. & Sakon, J. (2012). *Protein Sci.* **21**, 1554–1565. 10.1002/pro.2145 PMC3526996 22898990

[bb41] Philominathan, S. T., Matsushita, O., Gensure, R. & Sakon, J. (2009). *FEBS J.* **276**, 3589–3601. 10.1111/j.1742-4658.2009.07078.x PMC2782454 19490118

[bb42] Philominathan, S. T., Matsushita, O., Jordan, J. B. & Sakon, J. (2008). *Biomol. NMR Assign.* **2**, 127–129. 10.1007/s12104-008-9102-z PMC2718427 19636886

[bb43] Ponnapakkam, T., Katikaneni, R., Miller, E., Ponnapakkam, A., Hirofumi, S., Miyata, S., Suva, L. J., Sakon, J., Matsushita, O. & Gensure, R. C. (2011). *Calcif. Tissue Int.* **88**, 511–520. 10.1007/s00223-011-9485-1 21512758

[bb44] Ponnapakkam, T., Katikaneni, R., Nichols, T., Tobin, G., Sakon, J., Matsushita, O. & Gensure, R. C. (2011). *J. Endocrinol. Invest.* **34**, e392–e397. 10.3275/7864 21750397

[bb45] Ponnapakkam, T., Katikaneni, R., Sakon, J., Stratford, R. & Gensure, R. C. (2013). *Drug Discov. Today*, **19**, 204–208 10.1016/j.drudis.2013.07.015 PMC3979969 23932952

[bb46] Ponnapakkam, T., Katikaneni, R., Suda, H., Miyata, S., Matsushita, O., Sakon, J. & Gensure, R. C. (2012). *Calcif. Tissue Int.* **91**, 196–203. 10.1007/s00223-012-9626-1 PMC3693552 22806683

[bb47] Saito, W., Uchida, K., Ueno, M., Matsushita, O., Inoue, G., Nishi, N., Ogura, T., Hattori, S., Fujimaki, H., Tanaka, K. & Takaso, M. (2013). *J. Biomed. Mater. Res. A*, **102**, 3049–3055 10.1002/jbm.a.34974 24124060

[bb48] Schormann, N., Murrell, J. R., Liepnieks, J. J. & Benson, M. D. (1995). *Proc. Natl Acad. Sci. USA*, **92**, 9490–9494. 10.1073/pnas.92.21.9490 PMC40827 7568160

[bb49] Sides, C. R., Liyanage, R., Lay, J. O. Jr, Philominathan, S. T., Matsushita, O. & Sakon, J. (2012). *J. Am. Soc. Mass Spectrom.* **23**, 505–519. 10.1007/s13361-011-0309-3 PMC3389352 22207568

[bb50] The International Polycystic Kidney Disease Consortium (1995). *Cell*, **81**, 289–298. 10.1016/0092-8674(95)90339-9 7736581

[bb51] Thompson, J. D., Higgins, D. G. & Gibson, T. J. (1994). *Nucleic Acids Res.* **22**, 4673–4680. 10.1093/nar/22.22.4673 PMC308517 7984417

[bb52] Uchida, K., Matsushita, O., Naruse, K., Mima, T., Nishi, N., Hattori, S., Ogura, T., Inoue, G., Tanaka, K. & Takaso, M. (2013). *J. Biomed. Mater. Res. A*, **102**, 1737–1743. 10.1002/jbm.a.34841 PMC4232007 23775724

[bb53] Wang, Y.-K., Zhao, G.-Y., Li, Y., Chen, X.-L., Xie, B.-B., Su, H.-N., Lv, Y.-H., He, H.-L., Liu, H., Hu, J., Zhou, B.-C. & Zhang, Y.-Z. (2010). *J. Biol. Chem.* **285**, 14285–14291. 10.1074/jbc.M109.087023 PMC2863174 20207733

[bb54] Welgus, H. G., Jeffrey, J. J. & Eisen, A. Z. (1981*a*). *J. Biol. Chem.* **256**, 9511–9515. 6270089

[bb55] Welgus, H. G., Jeffrey, J. J. & Eisen, A. Z. (1981*b*). *J. Biol. Chem.* **256**, 9516–9521. 6270090

[bb56] Wilson, J. J., Matsushita, O., Okabe, A. & Sakon, J. (2003). *EMBO J.* **22**, 1743–1752. 10.1093/emboj/cdg172 PMC154464 12682007

[bb57] Yeats, C., Bentley, S. & Bateman, A. (2003). *BMC Microbiol.* **3**, 3. 10.1186/1471-2180-3-3 PMC151604 12625841

