# Supplementary material for: Structures of three polycystic kidney disease-like domains from Clostridium histolyticum collagenases ColG and ColH
Source: Acta Crystallogr D Biol Crystallogr. 2015 Feb 26;71(Pt 3):565–77. doi: 10.1107/S1399004714027722 (PMC4356367; doi:10.1107/S1399004714027722)
Supplement: Supplementary file 1 [file d-71-00565-sup1.pdf]

BIOLOGICAL
CRYSTALLOGRAPHY

Volume 71 (2015)

Supporting information for article:

Structures of three polycystic kidney disease-like domains from *Clostridium histolyticum* collagenase ColG and ColH

Ryan Bauer, Katarzyna Janowska, Kelly Taylor, Brad Jordan, Steve Gann, Tomasz Janowski, Ethan C. Latimer, Osamu Matsushita and Joshua Sakon

Table S1 Data collection and refinement statistics

	Apo s2 Form I
**Data Collection**	
X-ray wavelength (Å)	0.919
Space group	$P2_12_12_1$
a (Å), b (Å), c (Å)	45.0, 49.0, 70.9
β (°)	90.0
γ (°)	90.0
Resolution (Å)	40.3-1.6
Highest resolution bin (Å)	1.64-1.60
Number of reflections	165,013
Redundancies (a)	4.1 (3.6)
Completeness (%) (a)	99.9 (99.6)
I/σI (a)	30.9 (2.1)
R_{meas} (%) (a, b)	7.0 (69.2)
**Refinement**	
Unique reflections	20,159
R_{cryst} (%) (a, c)	16.2 (26.5)
R_{free} (%) 5% of data (a, d)	19.2 (30.6)
Average B-factor: Main chain A (Å^2)	11.2
Average B-factor: Side chain A (Å^2)	15.7
Average B-factor: Main chain B (Å^2)	11.6
Average B-factor: Side chain B (Å^2)	16.2
Average B-factor: Solvent (Å^2)	28.1
Ramachandran statistics	
Favored (%)	100
Additionally allowed (%)	0

Outliers (%)	0

(a) Data for the highest resolution shell are given in parenthesis

(b) $R_{meas} = \sum_h \sum_i \sqrt{\frac{N_h}{N_h - 1}} \left| I_{hi} - \mathrm{I_h} \right| / \sum_h \sum_i \mathrm{I_h}$

(c) $R_{cryst} = \sum_{hkl} \left| \left| F_{obs} \right| - \left| F_{calc} \right| \right| / \sum_{hkl} \left| F_{obs} \right|$ for the 95% of reflection data used for refinement.

(d) $R_{free} = \sum_{hkl} \left| \left| F_{obs} \right| - \left| F_{calc} \right| \right| / \sum_{hkl} \left| F_{obs} \right|$ for the 5% of reflection data excluded from refinement.

Table S2 Alternate conformations of the PKD-like domains

Domain	Molecule	Alternate conformations
Apo-s2a	Molecule A	S720 , K742, and S762
	Molecule B	D715 and K742
	Molecule C	K697, S720, and N732
	Molecule D	D715, S720, and N732
Holo-s2a	Molecule A	S686, S759, and T763
	Molecule B	S720 and S759
	Molecule C	None
	Molecule D	S762
	Molecule E	S686 and S762
	Molecule F	S759 and S762
	Molecule G	S686 and S759
	Molecule H	S686 and S762
Holo-s2b	Molecule A	K792, V793, S806, S822, S827, and M854
	Molecule B	S786, S806, S822, and S827
Apo-s2 Form I	Molecule A	R702, K717, R732, S736, T761, and S763
	Molecule B	I689, K691, S720, T730, T749, and T761
Apo-s2 Form II	Molecule A	E714, N747, T749, and T761
	Molecule B	K691, E714, V737, and S763

Table S3 Hydrogen bond totals for PKD-like domains in presence and absence of Ca^{2+}.

Domain	Molecule	NH…O	OH…O	NH…N	CH…O	total
Apo-s2a	Molecule A	45	8	19	95	167
	Molecule B	38	8	19	87	152
	Molecule C	37	7	18	93	155
	Molecule D	39	8	20	92	159
Holo-s2a	Molecule A	45	7	18	86	156
	Molecule B	43	6	18	90	157
	Molecule C	42	7	19	91	159
	Molecule D	46	6	18	88	158
	Molecule E	47	6	18	86	157
	Molecule F	41	7	18	95	161
	Molecule G	40	6	18	89	153
	Molecule H	40	7	18	84	149
Holo-s2b	Molecule A	45	12	20	73	150
	Molecule B	47	6	20	85	158
Apo-s2	Molecule A	46	5	18	77	146
	Molecule B	48	4	18	82	152
Holo-s2	Molecule A	45	6	17	70	138

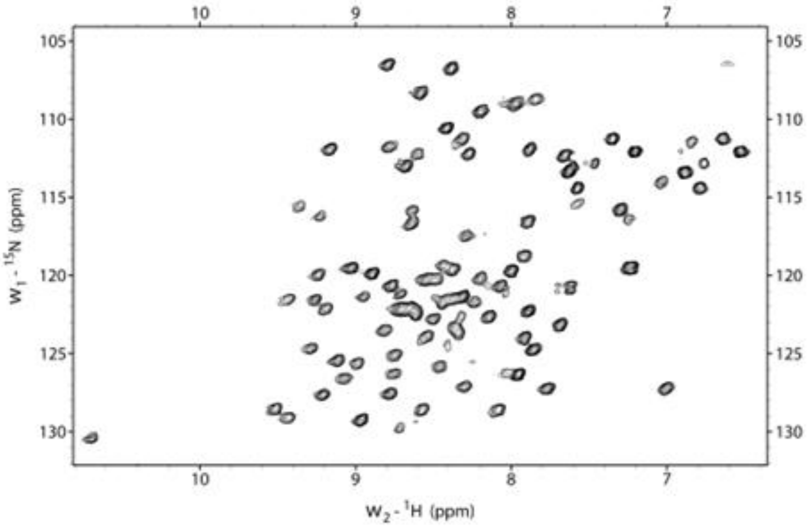

Figure S1 HSQC spectra for uniformly ^{15}N labeled s2. In the spectra, thirteen residues could not be identified due to extensive band broadening.

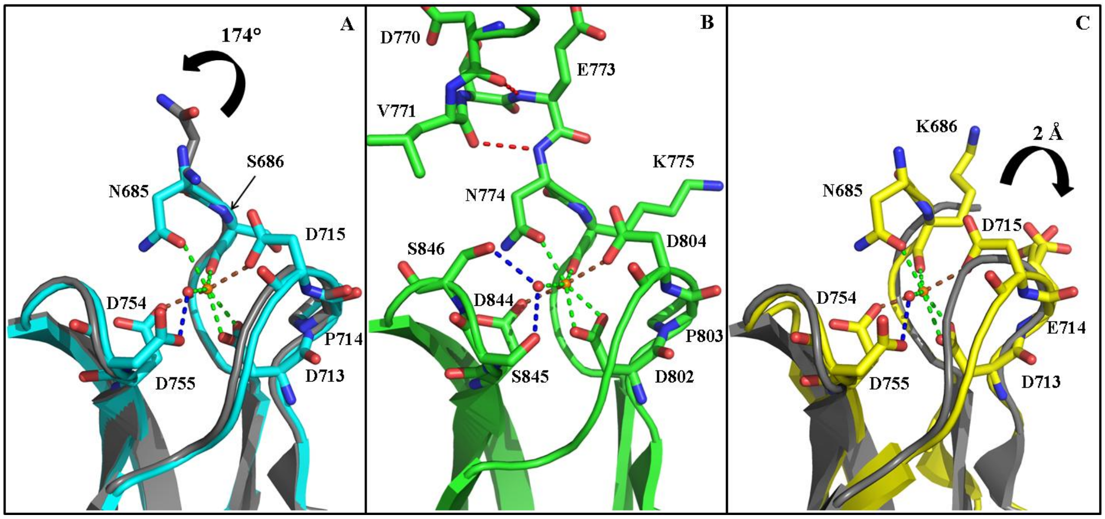

Figure S2 Ca^{2+}-induced structure rearraingment in the PKD-like domains. The N-terminal loop of s2a (A) is re-oriented as indicated by the rotation along the ψ bond of Asn685. The N-terminal linker of s2b (B) is observed in the crystal structure and indicates that the linker forms a 3_{10} helix (hydrogen bonding indicated by red dashes). Unlike s2a and s2b, the loop (713-717) of s2 (C) moves out to accommodate Ca^{2+}. This figure was prepared using PyMOL (Schrödinger, LLC.).

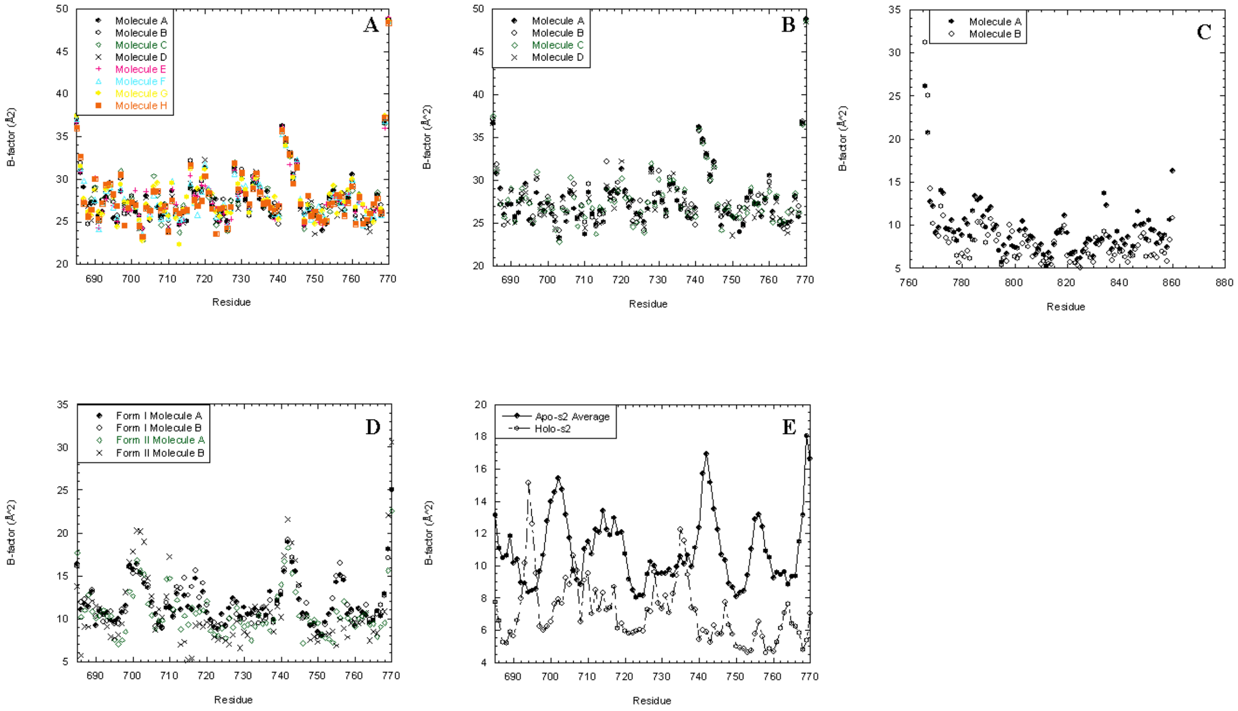

Figure S3 Per-residue B-factor trend for the PKD-like domains. The trends for *holo*-s2a (A), *apo*-s2a (B), *holo*-s2b (C), and *apo*-s2 (D) are similar. Comparison of the averaged B-factor of *apo*-s2 with *holo*-s2 (E) revealed the distinctly different influence Ca^{2+} has on potential dynamics.
